# Labile Calcium-Permeable AMPA Receptors Constitute New Glutamate Synapses Formed in Hypothalamic Neuroendocrine Cells during Salt Loading

**DOI:** 10.1523/ENEURO.0112-19.2019

**Published:** 2019-07-31

**Authors:** Shi Di, ZhiYing Jiang, Sen Wang, Laura M. Harrison, Eduardo Castro-Echeverry, Thomas C. Stuart, Marina E. Wolf, Jeffrey G. Tasker

**Affiliations:** 1 Department of Cell and Molecular Biology, Tulane University, New Orleans, LA 70118; 2 Tulane Brain Institute, Tulane University, New Orleans, LA 70118; 3 Department of Neuroscience, Rosalind Franklin University of Medicine and Science, North Chicago, IL 60064

**Keywords:** excitability, osmoregulation, oxytocin, paraventricular, supraoptic, vasopressin

## Abstract

Magnocellular neuroendocrine cells (MNCs) of the hypothalamus play a critical role in the regulation of fluid and electrolyte homeostasis. They undergo a dramatic structural and functional plasticity under sustained hyperosmotic conditions, including an increase in afferent glutamatergic synaptic innervation. We tested for a postulated increase in glutamate AMPA receptor expression and signaling in magnocellular neurons of the male rat hypothalamic supraoptic nucleus (SON) induced by chronic salt loading. While without effect on GluA1-4 subunit mRNA, salt loading with 2% saline for 5–7 d resulted in a selective increase in AMPA receptor GluA1 protein expression in the SON, with no change in GluA2-4 protein expression, suggesting an increase in the ratio of GluA1 to GluA2 subunits. Salt loading induced a corresponding increase in EPSCs in both oxytocin (OT) and vasopressin (VP) neurons, with properties characteristic of calcium-permeable AMPA receptor-mediated currents. Unexpectedly, the emergent AMPA synaptic currents were silenced by blocking protein synthesis and mammalian target of rapamycin (mTOR) activity in the slices, suggesting that the new glutamate synapses induced by salt loading require continuous dendritic protein synthesis for maintenance. These findings indicate that chronic salt loading leads to the induction of highly labile glutamate synapses in OT and VP neurons that are comprised of calcium-permeable homomeric GluA1 AMPA receptors. The glutamate-induced calcium influx via calcium-permeable AMPA receptors would be expected to play a key role in the induction and/or maintenance of activity-dependent synaptic plasticity that occurs in the magnocellular neurons during chronic osmotic stimulation.

## Visual Abstract

**Figure:**
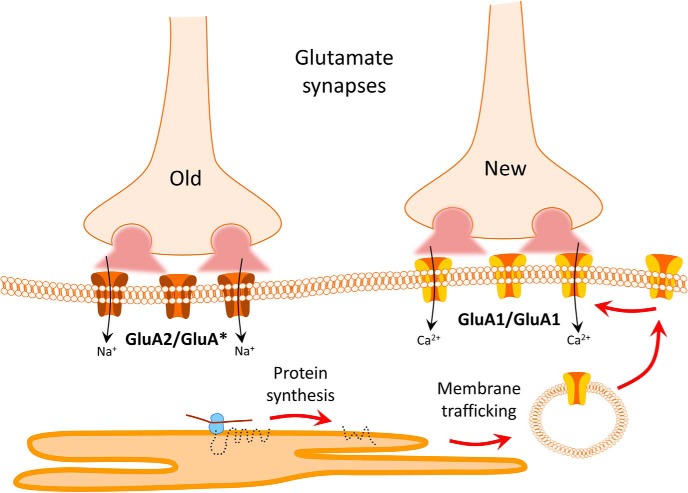

## Significance Statement

Oxytocin (OT)- and vasopressin (VP)-secreting neurons of the hypothalamus undergo robust synaptic plasticity during chronic osmotic stimulation, including an increase in excitatory synapses. Here, we show that chronic salt loading results in the emergence of calcium-permeable AMPA receptor-mediated synaptic currents in OT and VP neurons by inducing an increase in GluA1 AMPA receptor subunits without a coordinate increase in GluA2 subunits. The salt loading-induced calcium-permeable AMPA currents were rapidly silenced by inhibiting protein synthesis and mammalian target of rapamycin (mTOR) activity, suggesting that continuous dendritic protein synthesis is required to functionally maintain the new AMPA synapses. Calcium influx through calcium-permeable AMPA receptors should play a key role in the induction and/or maintenance of synaptic plasticity in OT and VP neurons by chronic osmotic stimulation.

## Introduction

Magnocellular neuroendocrine cells (MNCs) in the rat hypothalamic supraoptic nucleus (SON) and paraventricular nucleus (PVN) play a critical role in the regulation of blood pressure, blood volume and sodium balance. The primary mechanism by which the brain regulates systemic osmotic homeostasis is through the release of vasopressin (VP) and oxytocin (OT) from hypothalamic MNC axons in the pituitary (Brownstein et al., 1980; Verbalis, 1993; Gainer and Wray, 1994). Under acute hypertonic conditions, both VP and OT neurons are activated (Poulain and Wakerley, 1982) and rapidly release VP and OT from the posterior lobe of the pituitary into the systemic blood circulation, from where the hormones access the kidney to regulate water reabsorption and salt excretion (Verbalis, 1993). Chronic osmotic stimulation induced by salt loading induces hypertension in the rat (Choe et al., 2015). Salt loading causes dramatic morphologic restructuring of the SON and PVN that includes hypertrophy and decreased astrocytic coverage of the magnocellular neurons. It also leads to significant morphologic and functional synaptic plasticity, including increases in glutamatergic, GABAergic, and noradrenergic synaptic boutons on the magnocellular neurons, as well as increased glutamatergic signaling consistent with proliferation of glutamate release sites (Stern et al., 2000; Piet et al., 2002; Di and Tasker, 2004; Tasker et al., 2017) and enhanced glutamate receptor expression and/or function (Pak and Currás-Collazo, 2002).

Glutamate synapses make up a significant proportion of the synaptic innervation of the MNCs, representing 25% of all the SON synapses under basal conditions (Gribkoff and Dudek, 1988; van den Pol et al., 1990; Wuarin et al., 1992; Meeker et al., 1993, 1989). Glutamate AMPA receptors are tetramers comprised of combinations of four subunits, GluA1-4 (Borges and Dingledine, 1998; Dingledine et al., 1999). Expression of all four of the AMPA receptor subunits has been shown in the SON by immunohistochemistry and *in situ* hybridization (Nissen et al., 1994, 1995; Petralia et al., 1994; Hattori et al., 1998; Pak and Currás-Collazo, 2002). The ratio of GluA1-to-GluA2 subunits determines many of the biophysical properties of native glutamate receptors, including receptor kinetics, single-channel conductance, calcium permeability, and voltage-dependent sensitivity to endogenous polyamines (Washburn et al., 1997). Thus, the GluA1-to-GluA2 ratio is a major determinant of AMPA receptor function and is tightly regulated at the level of gene expression, RNA editing, receptor assembly, and receptor trafficking (Isaac et al., 2007).

The proliferation of glutamate synapses under conditions of chronic salt loading leads to increased excitatory synaptic innervation of the magnocellular neurons in the SON (Di and Tasker, 2004). Here, we postulated simply that the increase in functional excitatory innervation of these cells would be accompanied by a concomitant change in postsynaptic glutamate receptor expression and/or function. We combined quantitative polymerase chain reaction (qPCR), Western blotting, and membrane protein crosslinking analyses with whole-cell patch-clamp recordings in brain slices to test this hypothesis. Our findings revealed a more complex picture than anticipated, with newly formed glutamate synapses showing an apparent homomeric GluA1 AMPA receptor subunit composition and a highly labile expression that is dependent on continuous protein synthesis.

## Materials and Methods

### Animals

Adult male Sprague Dawley rats (Charles River) and VP-enhanced green fluorescent protein (eGFP) and OT-monomeric red fluorescent protein (RFP) Wistar transgenic rats were used in these studies (transgenic rats bred in-house, breeders kindly provided by Yoichi Ueta, Kitakyushu, Japan; Ueta et al., 2005; Katoh et al., 2011). All rats were group housed with *ad libitum* access to food and water, except for those undergoing salt loading. The salt-loading group was given access to only saline drinking water (2% NaCl, w/v) for 5–7 d; the euhydrated control group consisted of age-matched male rats receiving pure tap water. Animal weight was monitored and plasma osmolality was determined from trunk blood after decapitation. Animals in the salt-loaded group that did not show a marked increase in blood osmolality (≥320 mOsm/kg) were not included in the study. All experimental protocols were in accordance with United States Public Health Service guidelines and were approved by the Institutional Animal Care and Use Committees of Tulane University and the Rosalind Franklin University of Medicine and Science.

### Brain slice preparation

Rats were anesthetized with isoflurane inhalation (Sigma) and decapitated in a rodent guillotine, and the brains were removed and immersed for 1–2 min in chilled (0–1°C) HEPES buffer-based artificial CSF (aCSF) composed of the following: 140 mM NaCl, 3 mM KCl, 1.5 mM MgSO_4_, 1.4 mM NaH_2_PO_4_, 2.4 mM CaCl_2_, 11 mM glucose, and 5 mM HEPES, saturated with 100% O_2_. In some brain-slice experiments, we used a bicarbonate-based aCSF composed of the following: 124 mM NaCl, 2.7 mM KCl, 1.25 mM NaH_2_PO_4_, 26 mM NaHCO_3,_ 1.3 mM MgSO_4_, 2.5 mM CaCl_2_, 18 mM glucose, and 2.25 mM ascorbic acid, saturated with a 95% O_2_/5% CO_2_ gas mixture. The osmolarity was adjusted to 290–300 mOsm and the pH was adjusted to 7.2–7.4. The hypothalamus was blocked on ice and hypothalamic slices of 300-μm thickness were sectioned in cooled (0–2°C), oxygenated aCSF with a vibrating slicer (Vibratome Series 1000; Technical Products, Intl.).

### qPCR

**Figure 1.**
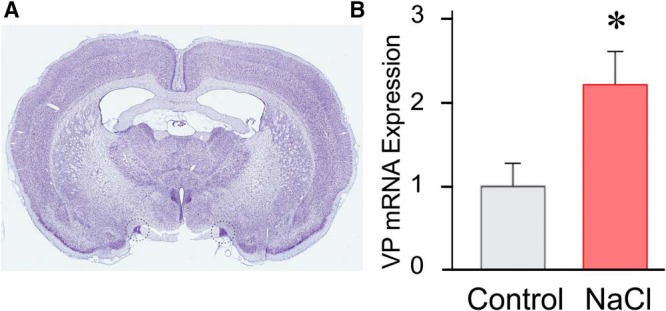
Effect of salt loading on VP mRNA expression in SON. ***A***, Image of a Nissl-stained coronal section of rat brain at the level of the hypothalamus showing the approximate locations of the 1-mm SON punches (dashed circles). ***B***, The relative VP mRNA expression measured by qPCR in SON punches from control (control) and salt-loaded (NaCl) rats, normalized to control; *p < 0.05.

For qPCR analyses, rats were anesthetized by isoflurane inhalation and then decapitated with a rodent guillotine. Whole brains were removed and quickly frozen in isopentane at –40°C and stored at –80°C. On the day of experiments, 1-mm-diameter tissue punches of the SON were made from 300-μm-thick slices with a Miltex Biopsy Punch (Fig. 1*A*). Tissue samples were homogenized and RNA was isolated using the Aurum Total RNA Mini kit (Bio-Rad). An on-column DNase digestion step was performed to eliminate genomic DNA. RNA was then quantified with an Implen NanoPhotometer, and the RNA integrity was determined by agarose gel electrophoresis, which indicated intact RNA for all samples, without genomic DNA contamination. RNA (400 ng) was then reverse-transcribed with iScript Reverse Transcription Supermix (Bio-Rad) using random and oligo dT primers, and the cDNA was stored at –20°C. Negative control reactions were performed in the absence of reverse transcriptase enzyme. PCR with iTaq Universal SYBR Green Supermix (Bio-Rad) was performed in triplicate on a CFX thermocycler (Bio-Rad, 40 cycles). For each primer pair, a temperature gradient with annealing temperatures ranging from 55–70°C was tested, and 60°C was used for subsequent PCR reactions. The linear range and the efficiency were determined by performing a dilution curve for each primer pair consisting of eight 10-fold dilutions. Efficiencies ranged from 100–110%. PCR primer sequences and product sizes are shown in Table 1. Melt curves and agarose gel electrophoresis demonstrated single products for each reaction at the expected size. Data were analyzed by the 2^-ΔΔC^_T_ method. The geometric mean of cytochrome C-1, cyclophilin, and GAPDH was used to normalize gene expression.

**Table 1.** Primer pairs used for qPCR analysis

Gene	Accession number	Primer sequence (5’ to 3’)	Product size (bp)	Reference
VP	M25646	Forward: GCTGCTGCTTAGGCTGGTACA Reverse: GGCGATGGCTCAGTAGACCC	82	Primer3
GluA1	NM_031608	Forward: GTGTCTTCTCCTTTCTTGACCCTTT Reverse: CTCTTCGCTGTGCCATTCGTAG	128	Primer3
GluA2	NM_017261	Forward: AGAGAAAGAATACCCTGGAGCACA Reverse: TCATCACTTGGACAGCATCATACG	80	Primer3
GluA3	NM_032990	Forward: ATTCAGCAAATAGACCCATCTTAGC Reverse: ATCACCACCAACAGGAAATACCAAA	148	Primer3
GluA4	NM_017263	Forward: AAACAGGCAAAGATAGGAGAAAGGG Reverse: TCAGAGAGGCATTGAAGACGATGG	129	Primer3
GAPDH	X02231	Forward: ACATGCCGCCTGGAGAAACCT Reverse: GCCCAGGATGCCCTTTAGTGG	90	Kindlundh-Högberg et al. (2008)
Cytochrome C-1	NM_001277194	Forward: CTCTCCTCCTTGGACCACAC Reverse: CGGTAAGCCACATAATCCA	95	Dachtler et al. (2014)^*^
Cyclophilin	M19533	Forward: GAGCGTTTTGGGTCCAGGAAT Reverse: AATGCCCGCAAGTCAAAGAAA	90	Kindlundh-Högberg et al. (2008)

* Adapted for rat.

### Western blot

For Western blot analyses, rats were deeply anesthetized with isoflurane, 300-μm-thick brain slices were prepared as above, and 1-mm-diameter tissue punches of the SON were made with a Miltex Biopsy Punch using the optic chiasm/tracts as landmarks (Fig. 1). The SON tissue samples were added directly to lysis buffer (20 mM Tris, pH 8.0, 137 nM NaCl, 10% glycerol, 1% Nonidet P-40, and protease inhibitor cocktail) and homogenized. Lysates were clarified by centrifugation and protein was determined via a modified Lowry assay (DC Protein Assay, Bio-Rad). Samples (20-µg total protein/lysate) were loaded onto Any kDa TGX Stain-Free gels (Bio-Rad) and electrophoresed under reducing conditions. Proteins were then transferred onto polyvinylidene fluoride membranes for immunoblotting. Membranes were blocked with 5% nonfat dry milk in TBS-Tween 20 (TBS-T) at pH 7.4 for 1 h at room temperature, followed by incubation with rabbit polyclonal antibody for GluA1 (1:500, AB1504; Millipore/Sigma), or GluA4 (1:500, AB1508; Millipore), or mouse monoclonal antibody for GluA2 (1:1000, MABN1189; Millipore/Sigma) or GluA3 (1:1000; MAB5416, Millipore) overnight at 4°C. Membranes were then washed extensively with TBS-T solution, incubated for 60 min with HRP-conjugated anti-rabbit IgG or anti-mouse IgG (1:1000; Cell Signaling Technologies, Danvers, MA or 1:10 000; Millipore Cell Signaling, Lake Placid, NY), and washed extensively again in the TBS-T solution. Following incubation in chemiluminescence detecting substrate (Clarity Western ECL Substrate, Bio-Rad), membranes were imaged on a ChemiDoc Imaging System (Bio-Rad). Image Lab software (Bio-Rad) was used to determine optical densities of individual bands, which were normalized to total protein on the blot.

### Membrane protein crosslinking

Surface and intracellular levels of AMPA receptor subunits were quantified using a protein crosslinking method described previously (Boudreau et al., 2012). Briefly, cell surface-expressed proteins are covalently crosslinked using the membrane-impermeable, bifunctional crosslinker bis(sulfosuccinimidyl)suberate (BS^3^). This increases the apparent molecular weight of crosslinked surface receptors recognized by the antibody, while intracellular receptors are not modified. Thus, surface and intracellular receptor pools can be separated and quantified using SDS-PAGE and immunoblotting. Brain slices prepared as described above were diced on a McIlwain tissue chopper and were added to sample tubes containing 1 ml of ice-cold aCSF, to which BS^3^ (Pierce) had been added just before the chopped tissue samples at a final concentration of 2 mM. Samples were incubated at 4°C for 30 min with gentle mixing. The crosslinking reaction was terminated by adding 100 μl of 1 M glycine stock solution, for a final glycine concentration of 100 mM, and then incubated at 4°C for 10 min with gentle mixing. The chopped tissue samples were then pelleted by centrifugation (2 min at 20,000 × g) and the supernatant was discarded. Pellets were resuspended in 150-μl ice-cold lysis buffer containing protease and phosphatase inhibitors [25 mM (pH 7.4) HEPES, 500 mM NaCl, 2 mM EDTA, 1 mM DTT, 1 mM phenylmethyl sulfonyl fluoride (PMSF), 20 mM NaF, 0.1% Nonidet P-40 (v/v), and 1× protease inhibitor cocktail] and homogenized rapidly by sonicating for 5 s. Samples were then spun at 20,000 × *g* for 2 min and immediately placed on ice, aliquoted and stored at –80°C until Western blot analysis was performed with β-actin as a loading control.

### Electrophysiology

Brain slices were bisected down the midline and hemi-slices were incubated in warmed aCSF (32–34°C) for recovery (20 min), then in oxygenated aCSF at room temperature for at least 1 h before recording. A single hemi-slice at a time was transferred to a submersion recording chamber mounted on a fixed-stage upright microscope (Olympus BX51WI) and continuously superfused at 2 ml/min with oxygenated aCSF at 31–32°C.

Patch pipettes were pulled from borosilicate glass (1.65 mm outer diameter, 1.2 mm inner diameter; KG33; King Precision Glass) on a horizontal micropipette puller (Flaming/Brown P-97, Sutter Instruments) to a resistance of 3–5 MΩ, and were filled with a solution containing the following: 130 mM K-gluconate, 1 mM NaCl, 1 mM MgCl_2_, 10 mM EGTA, 2 mM Mg-ATP, 0.5 mM Na-GTP, 1 mM CaCl_2_, and 10 mM HEPES. To study the current−voltage relations of AMPA receptor-mediated synaptic currents, a Cs-based, spermine–containing patch solution was used, composed of the following: 140 mM CsCl, 2 mM MgCl_2_, 10 mM HEPES, 2 mM Mg-ATP, 0.3 mM Na-GTP, 2 mM QX314, and 0.1 mM spermine. The patch solution pH was adjusted to 7.3 and the osmolarity was adjusted to 300 mOsm.

Following transfer to the recording chamber, hemi-slices were allowed to equilibrate for at least 15 min before the start of recordings. SON neurons were visualized on a video monitor using a cooled CCD camera; infrared illumination and differential interference contrast (IR/DIC) optics, and were patch-clamped under visual control. After achieving the whole-cell configuration, series resistance and whole-cell capacitance were adjusted and monitored continuously throughout experiments. Magnocellular neurons were identified based on their location in the SON and their electrophysiological properties (i.e., robust A-type K^+^ current and/or transient outward rectification (Tasker and Dudek, 1991; Luther et al., 2002). Recordings were performed in SON neurons from VP-eGFP and OT-RFP rats, in which VP-expressing neurons and OT-expressing neurons were identified, respectively, by their eGFP or RFP fluorescence under epifluorescence illumination before switching to IR/DIC to obtain patch-clamp recordings. Patch-clamp recordings were performed using a Multiclamp 700A or 700B amplifier (Molecular Devices). Data were low-pass filtered at 2 kHz with the amplifier and sampled at 10 kHz using the pClamp 9 or 10 suite of software (Molecular Devices).

Slices were bathed in normal aCSF with picrotoxin (50 µM) or bicuculline (10 µM) to block GABA_A_ receptor-mediated synaptic currents and DL-2-amino-5-phosphonopentanoic acid (AP5; 40 µM) to block NMDA receptor-mediated currents. Changes in spontaneous EPSC (sEPSC) frequency, amplitude and decay time and evoked EPSC (eEPSC) amplitude and paired-pulse ratio were analyzed. The eEPSCs were elicited by extracellular electrical stimulation at 0.1 Hz at 50–70% of maximal amplitude via a concentric stimulating electrode (FHC) placed dorsal-medial to the SON. The paired-pulse stimulation paradigm was administered using a pair of identical electrical stimuli delivered at a 45-ms interval. We also recorded eEPSCs at holding potentials of –60 mV and +40 mV and with 20-mV incremented steps to calculate changes in inward rectification and to establish a current–voltage relationship, respectively. Averages of six individual eEPSC responses at each holding potential were used for analyses. I-V curves were normalized to the current response at –60 mV for comparison. The rectification index was determined by taking the ratio of the peak EPSC amplitudes recorded at Vm = –60 mV to those recorded at Vm = +40 mV. Changes in capacitance, membrane resistance and series resistance were monitored continuously throughout the experiments. The series resistance at the beginning of recordings was <20 MΩ and recordings were discarded if changes >20% occurred during the recordings.

### Drug application

The drugs used in electrophysiology experiments included the GABA_A_ receptor antagonists picrotoxin (50 µM) and bicuculline (10 µM) to block inhibitory postsynaptic currents and the NMDA receptor antagonist AP5 (40 µM) to isolate AMPA receptor-dependent EPSCs. 1-Naphthyl-acetyl-spermine (NAS; Hello Bio) was used to selectively inhibit GluA2-lacking/calcium-permeable AMPA receptor-mediated synaptic currents. The specific inhibitor of mammalian target of rapamycin (mTOR) rapamycin (Rapa; 500 nM), the selective inhibitor of eukaryotic protein synthesis cycloheximide (CHX; 25 µM) and the gene transcription inhibitor actinomycin D (ACT; 25 µM) were applied extracellularly in the aCSF to preincubate the brain slices, and/or intracellularly during recordings via the patch pipette. All drugs, unless specified, were purchased from Tocris Bioscience.

### Statistical analyses

Statistical analyses of data were performed using the Student’s paired *t* test for within-group comparisons and the student’s unpaired *t* test for between-group comparisons. A one-way or two-way ANOVA was used for multiple comparisons, followed by *post hoc* multiple comparisons with the Tukey’s or Dunnett’s test where appropriate. The significance threshold for all analyses was set at *p* < 0.05. Statistical analyses were performed with Excel (Microsoft), Sigmaplot (Systat Software Inc.), or Prism (GraphPad Software). Data are expressed as the mean ± SEM.

## Results

Body weights were measured immediately before placing the salt-loaded animals on the 2% saline solution and the control animals on pure water and again after 5–7 d of saline or water consumption. Representative cohorts of control animals gained an average of 39% (*n* = 8) and salt-loaded animals lost an average of 11% of their body weight (*n* = 10) by the end of the saline/water consumption period. Plasma osmolality measured at the end of the saline/water consumption period was 294.5 ± 1.3 mOsm/kg in six control rats and 353.4 ± 5.6 mOsm/kg in 19 salt-loaded rats (unpaired *t* test, *t*_(23)_ = 5.6, *p* < 0.001).

### VP mRNA expression with salt loading

Previous studies reported that VP mRNA expression undergoes a 1.5- to 2-fold increase with salt loading (Van Tol et al., 1987; Sherman et al., 1988; Arnauld et al., 1993). Here, we used qPCR to test for changes in VP mRNA expression with salt loading and to serve as a positive control for our qPCR analyses. Salt loading caused a 2.2-fold increase in VP mRNA expression in SON samples (*t*_(16)_ = 2.4, *p* < 0.05; Fig. 1).

### AMPA receptor subunit mRNA expression

**Figure 2.**
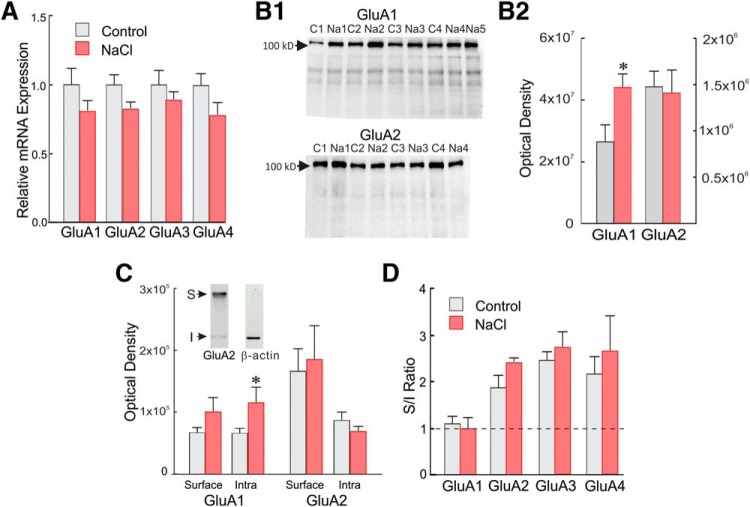
Effects of chronic salt loading on AMPA receptor subunit expression in the SON. ***A***, qPCR of AMPA receptor subunit mRNA expression in SON punches from control and salt-loaded (NaCl) rats revealed no significant change in any of the AMPA receptor subunit mRNAs, GluA1-4, with salt loading. ***B1***, Representative Western blots of GluA1 and GluA2 total protein expression in SON punches from four control rats (C1–C4) and five (GluA1: Na1–Na5) or four (GluA2: Na1–Na4) salt-loaded rats. ***B2***, Mean optical density measurements of GluA1 and GluA2 protein bands from SON tissue from control (*N* = 8) and salt-loaded (NaCl, *N* = 7) rats revealed an increase in total GluA1 protein with salt loading. ***C***, Surface and intracellular GluA1 and GluA2 protein levels in SON punches from control (*N* = 7, 8, respectively) and salt-loaded (NaCl, *N* = 5, 7, respectively) rats determined by Western blot following BS^3^ surface protein crosslinking. Inset, Representative Western blots of SON tissue from a control rat treated with the membrane impermeant crosslinking agent BS^3^ and probed with antisera to GluA2 and β-actin. Two GluA2 bands were detected: a high molecular weight band representing the crosslinked surface protein aggregate (S) and a band at the predicted molecular weight for GluA2 (∼100 kDa) that represents the unmodified intracellular pool (I). β-Actin served as an intracellular control and was found at its predicted molecular weight (∼40 kDa), confirming the lack of crosslinking of intracellular proteins. ***D***, Surface-to-intracellular (S/I) protein ratios determined following BS^3^ surface protein crosslinking for GluA1-GluA4 AMPA receptor subunits in SON punches from control and salt-loaded (NaCl) rats. Despite the increase in total GluA1 protein, there was no change in the subunit S/I ratios between SON tissues from control and salt-loaded rats for any of the AMPA receptor subunits; *p < 0.05.

To determine whether the changes in glutamate synapse number and glutamate responses induced by chronic salt loading (Di and Tasker, 2004; Bonfanti and Theodosis, 2009) are accompanied by changes in AMPA receptor expression, we first conducted a qPCR analysis to test for an increase in the total expression of AMPA receptor subunit mRNA in chronically salt-loaded rats (*n* = 9) compared to control rats (*n* = 9). Our results confirmed previous findings that the mRNAs of each of the four AMPA receptor subunits are expressed in the SON (Petralia and Wenthold, 1992; Martin et al., 1993; Sato et al., 1993; Watanabe et al., 1993; van den Pol et al., 1994; Ginsberg et al., 1995; Al-Ghoul et al., 1997). Unexpectedly, there was no significant change in the mRNA expression of any of the AMPA receptor subunits with salt loading (unpaired *t* test; GluA1: *t*_(16)_ = 1.3, *p* = 0.21; GluA2: *t*_(16)_ = 1.8, *p* = 0.09; GluA3: *t*_(16)_ = 0.95, *p* = 0.36; GluA4: *t*_(16)_ = 1.4, *p* = 0.19; Fig. 2*A*).

The finding that none of the AMPA receptor subunits showed a significant increase in mRNA expression with salt loading suggested that the hypothesized upregulation of the glutamate receptor expression, if it occurs, must manifest at a post-transcriptional stage. We used a Western blot approach to test for this possibility of plasticity in protein expression of the AMPA receptor subunits with chronic salt loading.

### AMPA receptor subunit protein expression

We measured AMPA receptor subunit protein levels in SON tissue homogenates from control (*n* = 8) and salt-loaded rats (*n* = 7). In response to salt loading, GluA1 was the only subunit that showed a significant change in protein level, increasing by 69% (unpaired *t* test, *t*_(13)_ = 2.484, *p* < 0.05; Fig. 2*B*). The GluA2 subunit protein level did not change (*t*_(13)_ = 0.236, *p* = 0.817; Fig. 2*B*). The GluA3 and GluA4 protein levels also did not change (*p* > 0.05; data not shown).

We next used a surface protein crosslinking approach (Boudreau et al., 2012) to compare cell surface versus intracellular pools of GluA1-GluA4 protein in SON samples. Using Western blot analysis of crosslinked surface protein and unmodified intracellular protein, we found that both the surface and intracellular GluA1 protein levels increased in the salt-loaded animals (51% and 73% increase, respectively, compared to controls), although the increase in intracellular GluA1 did not reach statistical significance (*p* = 0.137; Fig. 2*C*). The surface and intracellular GluA2 protein levels did not change with salt loading (Fig. 2*C*), nor did the GluA3 and GluA4 protein levels (data not shown). Despite the change in total GluA1 protein, the relative membrane localization (i.e., S/I ratio) of GluA1, as well as the other three AMPA receptor subunits, remained unchanged (Fig. 2*D*). Taken together with the increase in total GluA1 protein (Fig. 2*B*) and the lack of alteration in any of the subunit mRNAs (Fig. 2*A*), these results indicate a selective posttranscriptional increase in GluA1 protein in the SON after salt loading that is distributed proportionately between the intracellular and membrane compartments. This suggested an increase in the membrane GluA1-to-GluA2 AMPA receptor subunit ratio in SON neurons with salt loading, which should have significant effects on AMPA receptor signaling. We tested for this possibility using whole-cell patch-clamp recordings of SON magnocellular neurons in brain slices.

### Changes in excitatory synaptic inputs

**Figure 3.**
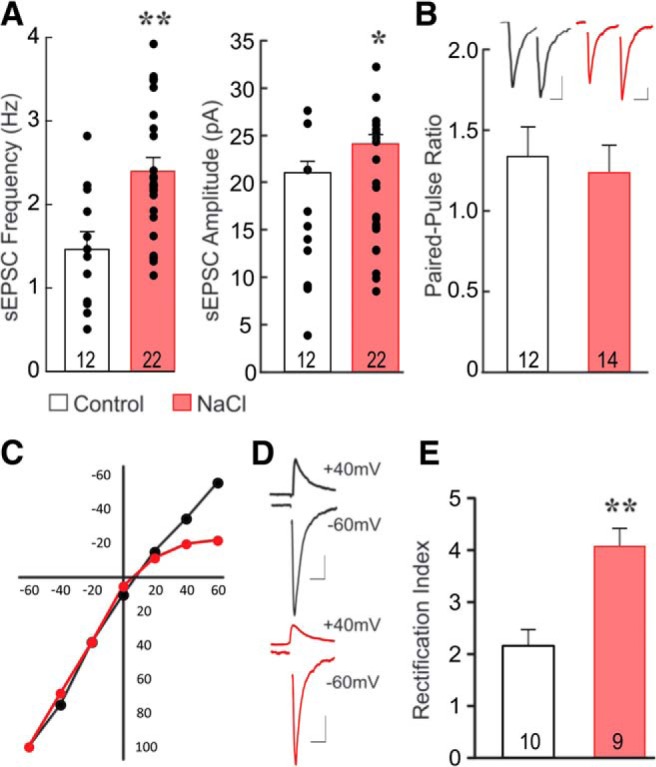
Increase in inwardly rectifying AMPA receptor signaling following salt loading. ***A***, Scatter plots of individual neuronal means and group means of sEPSC frequencies and amplitudes. Identified VP neurons recorded in slices from salt-loaded VP-eGFP rats (NaCl) showed a significant increase in the mean sEPSC frequency and amplitude compared to the control group. ***B***, The paired-pulse ratio of eEPSCs (inset) was unchanged following salt loading, suggesting that the increased glutamatergic synaptic activity was not due to a change in the probability of glutamate release. ***C***, The eEPSC current−voltage relationship showed inward rectification at Vm > 0 mV in SON neurons from salt-loaded rats (red curve, *n* = 6) but not controls (black curve, *n* = 4). The mean I-V curves are group averages of normalized individual I-V curves, expressed as percent of eEPSC recorded at −60 mV. ***D***, Representative AMPA receptor-mediated eEPSC responses recorded at holding potentials of –60 mV and +40 mV in SON neurons from control and salt-loaded rats. ***E***, The rectification index (eEPSC amplitude at –60 mV/eEPSC amplitude at +40 mV) increased in SON neurons from salt-loaded rats, suggesting the emergence of calcium-permeable AMPA receptor-containing glutamate synapses with salt loading. VP and OT neurons were pooled together for panels ***B–E***. Numerals in bars represent recorded cell numbers; *p < 0.05, **p < 0.01. Scale bars = 100 pA, 20 ms.

SON neurons recorded in slices from salt-loaded rats showed an increase in the frequency and amplitude of sEPSCs compared with SON neurons from control rats. In recordings of identified VP neurons in slices from VP-eGFP rats, there was an increase of 60% in the mean sEPSC frequency, from 1.5 ± 0.2 to 2.4 ± 0.2 Hz (unpaired *t* test, *t*_(32)_ = 3.3, *p* < 0.001), and an increase of 14% in the mean sEPSC amplitude, from 21.1 ± 1.2 to 24.1 ± 1.0 pA (*t*_(32)_ = 2.1, *p* < 0.05), in VP neurons from salt-loaded rats compared to control rats (Fig. 3*A*). Similar findings were obtained from identified OT neurons recorded in slices from OT-RFP rats, which showed a 93% increase in sEPSC frequency, from 1.5 ± 0.2 to 2.9 ± 0.6 Hz (*t*_(10)_ = 2.5, *p* < 0.05), and a 20% increase in the sEPSC amplitude, from 19.6 ± 1.1 to 23.5 ± 1.1 pA (*t*_(10)_ = 2.4, *p* < 0.05), in slices from salt-loaded compared to control rats.

The increase in sEPSC frequency in SON neurons following salt loading could be due to an increase in the probability of glutamate release from existing synapses or to an increase in the number of glutamatergic synapses. To determine whether the increase in glutamatergic synaptic inputs was caused by an increase in the probability of glutamate release, we performed a paired-pulse analysis on eEPSCs. The paired-pulse ratio did not differ between neurons from the control and salt-loaded groups (Fig. 3*B*). Thus, the paired/pulse ratios in SON neurons from control and salt-loaded rats were 1.3 ± 0.2 and 1.2 ± 0.2, respectively, in VP neurons from VP-eGFP rats (unpaired *t* test, *t*_(16)_ = 1.01, *p* = 0.32) and 1.3 ± 0.2 and 1.1 ± 0.1, respectively, in OT neurons from OT-RFP rats (*t*_(6)_ = 1.06, *p* = 0.33). These findings suggest that the increase in sEPSC frequency is not caused by an increase in the probability of glutamate release, but is due rather to an increased number of glutamate release sites. There were no differences in the sEPSC and paired-pulse measures between identified VP and OT neurons, so VP and OT neurons were pooled in subsequent analyses, unless otherwise stated.

The increase in GluA1 surface and intracellular protein with no change in GluA1 mRNA or GluA2, GluA3 or GluA4 protein or mRNA expression suggested a post-transcriptional increase in the GluA1-to-GluA2 ratio. GluA2-lacking AMPA receptors are calcium-permeable and generate synaptic currents that display a voltage-dependent inward rectification (Hollmann et al., 1991). We next determined the contribution of GluA2-lacking AMPA receptors to glutamatergic synaptic transmission in magnocellular neurons in the SON by analyzing the current−voltage relations of AMPA receptor-dependent eEPSCs. We recorded the AMPA receptor-mediated eEPSCs at holding potentials (Vms) of –60 and +40 mV and calculated the AMPA current rectification index as the peak eEPSC amplitude at –60 mV/the peak eEPSC amplitude at +40 mV. In a subset of neurons, we recorded AMPA receptor-mediated eEPSCs at Vms ranging from –60 to +40 mV in 20-mV incremental steps to generate current−voltage curves for the synaptic AMPA current. The current–voltage relationship of AMPA eEPSCs was nearly linear in SON neurons from control rats, suggesting little or no contribution of rectifying AMPA receptors to glutamatergic synaptic transmission in SON neurons under control conditions, but showed a significant inward rectification at Vm > 0 mV in SON neurons from salt-loaded rats (Fig. 3*C*). The rectification index was increased from 2.2 ± 0.3 in SON neurons from control rats to 4.0 ± 0.3 in SON neurons from salt-loaded rats (unpaired *t* test, *t*_(17)_ = 4.1, *p* < 0.001; Fig. 3*D*,*E*). This indicated that there is a significant increase in the rectifying AMPA receptor contribution to excitatory synaptic currents in SON neurons following chronic salt loading, suggesting an increase in calcium-permeable AMPA receptors.

**Figure 4.**
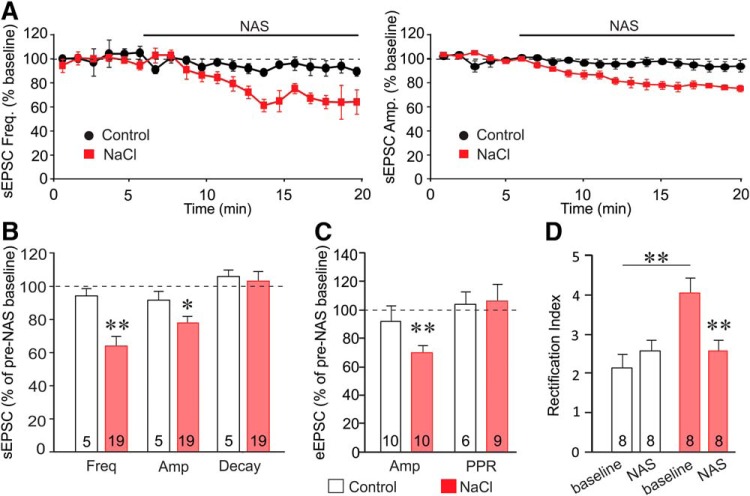
Change in EPSC sensitivity to the calcium-permeable AMPA receptor antagonist NAS following salt loading. ***A***, NAS reduced the mean frequency (left) and amplitude (right) of sEPSCs over time in SON neurons from salt-loaded (NaCl) rats, but not in SON neurons from control rats. ***B***, The effect of NAS on mean sEPSC frequency (Freq), amplitude (Amp), and decay time (Decay) in SON neurons from salt-loaded rats (NaCl) and control rats, expressed as a percentage of baseline. NAS decreased the sEPSC frequency and amplitude in SON neurons from salt-loaded rats but not control rats. ***C***, NAS caused a decrease in the mean amplitude of eEPSCs in SON neurons from salt-loaded rats, but not control rats. NAS had no effect on the paired-pulse ratio of eEPSCs (PPR) in SON neurons from either control or salt-loaded rats. ***D***, NAS had no effect on the eEPSC rectification index in SON neurons from control rats, but reversed the increase in the rectification index in SON neurons from salt-loaded rats. Numerals in bars represent recorded cell numbers; *p < 0.05, **p < 0.01 compared to control or baseline.

We next examined the effect of the selective antagonist of GluA2-lacking, calcium-permeable AMPA receptors NAS on both sEPSCs and eEPSCs in SON magnocellular neurons from control and salt-loaded rats. NAS (100 μM) had no effect on sEPSCs and eEPSCs in SON neurons from control rats, but caused a robust suppression of sEPSCs and eEPSCs in SON neurons in slices from salt-loaded rats. Thus, bath application of NAS was without effect on the frequency (94% of baseline, paired *t* test, *t*_(4)_ = 1.27, *p* = 0.27) or amplitude (94% of baseline, *t*_(4)_ = 0.91, *p* = 0.39) of sEPSCs in SON neurons from control rats, but caused a significant decrease in the frequency (63% of baseline, paired *t* test, *t*_(18)_ = 5.6, *p* < 0.001) and amplitude (80% of baseline, *t*_(18)_ = 3.3, *p* < 0.05) of sEPSCs in SON neurons from salt-loaded rats (Fig. 4*A*,*B*). Similarly, NAS caused a non-significant decrease in the eEPSC amplitude in SON neurons from control rats (94% of baseline, paired *t* test, *t*_(9)_ = 2.1, *p* = 0.06), but caused a robust decrease in the eEPSC amplitude in neurons from salt-loaded rats (68% of baseline, *t*_(9)_ = 3.9, *p* = 0.008; Fig. 4*C*). NAS had no effect on the paired-pulse ratios in the SON neurons from either the control or the salt-loaded group (paired *t* test, *p* > 0.05; Fig. 4*C*), indicating again that the salt loading-induced synaptic plasticity is not due to an increase in the probability of presynaptic glutamate release.

Current−voltage analysis of eEPSCs during NAS application revealed a loss of the inward rectification of the AMPA currents in SON neurons from salt-loaded rats (one-way ANOVA, *F*_(3,28)_ = 7.67, *p* < 0.001). Thus, NAS reversed the increase in the eEPSC rectification index from 4.1 ± 0.4 to 2.6 ± 0.2 (*post hoc* Tukey’s test, *p* < 0.01) in SON neurons from salt-loaded rats, while it was without effect on the eEPSC rectification index in SON neurons from control rats (*post hoc* Tukey’s test, *p* = 0.519; Fig. 4*D*). The increase in the sensitivity of EPSCs to NAS and the NAS-induced reversal of the inward rectification of EPSCs in SON neurons from salt-loaded rats suggest that there is an induction of calcium-permeable AMPA receptors at glutamate synapses by salt loading, which is consistent with the increase in the GluA1-to-GluA2 ratio of the AMPA receptors following salt loading. The linearity of the AMPA current−voltage relationship and the insensitivity of the AMPA currents to NAS in SON neurons from control rats suggest a relative lack of calcium-permeable AMPA receptors at glutamate synapses under control osmotic conditions.

**Figure 5.**
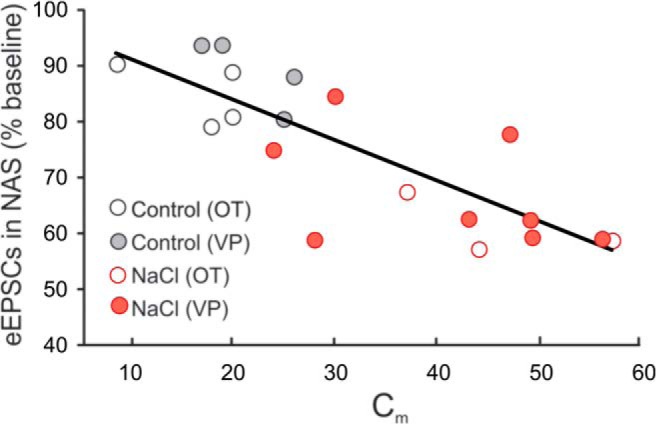
Correlation of the membrane surface area to NAS sensitivity. There was a significant correlation between the increase in magnocellular neuron surface area, as measured by membrane capacitance (C_m_), and the change in eEPSC amplitude in NAS.

If the salt loading-induced plasticity is not due to an increase in the probability of glutamate release, then the modulation of sEPSC frequency by NAS is likely to be a postsynaptic effect, since spontaneous excitatory synaptic activity in magnocellular neurons is generated almost entirely by spike-independent glutamate release (Kombian et al., 2000; Boudaba et al., 2003; Hirasawa et al., 2004). If this is the case, then the reduction in sEPSC frequency caused by blocking calcium-permeable AMPA receptors in magnocellular neurons from salt-loaded rats, but not in neurons from control rats, suggests silencing of the newly formed excitatory synapses. The new glutamate synapses formed during salt loading appear, then, to be composed of homomeric GluA1, calcium-permeable AMPA receptors, whereas the “old” synapses in neurons from the dehydrated rats and nearly all the synapses in neurons from control rats are composed of GluA2-containing, calcium-impermeable AMPA receptors. If the new synapses added following salt loading are dependent on the new surface area added by hypertrophy of the magnocellular neurons (Theodosis et al., 1998), and only the new synapses are composed of calcium-permeable AMPA receptors, then the increased sensitivity of the eEPSCs to the calcium-permeable AMPA-receptor antagonist should be tightly correlated to the increase in magnocellular neuron surface area. To test this, we performed a Pearson correlation between the NAS effect on eEPSCs and the cell surface area of recorded cells, measured as membrane capacitance, in oxytocinergic and vasopressinergic SON neurons from control and salt-loaded rats. There was a highly significant correlation between membrane capacitance and NAS sensitivity of the eEPSCs in the SON neurons (correlation coefficient = 0.8, *n* = 19, *p* < 0.001; Fig. 5). A significant correlation was also observed between the membrane capacitance and the NAS sensitivity of the sEPSCs (correlation coefficient = 0.3, *n* = 23, *p* < 0.01). The correlation between the increase in surface area and the relative increase in the NAS sensitivity of EPSCs in SON neurons from salt-loaded rats suggests that most of the additional glutamate synapses that were newly formed on the expanded membrane surface during salt loading are composed of calcium-permeable AMPA receptors.

**Figure 6.**
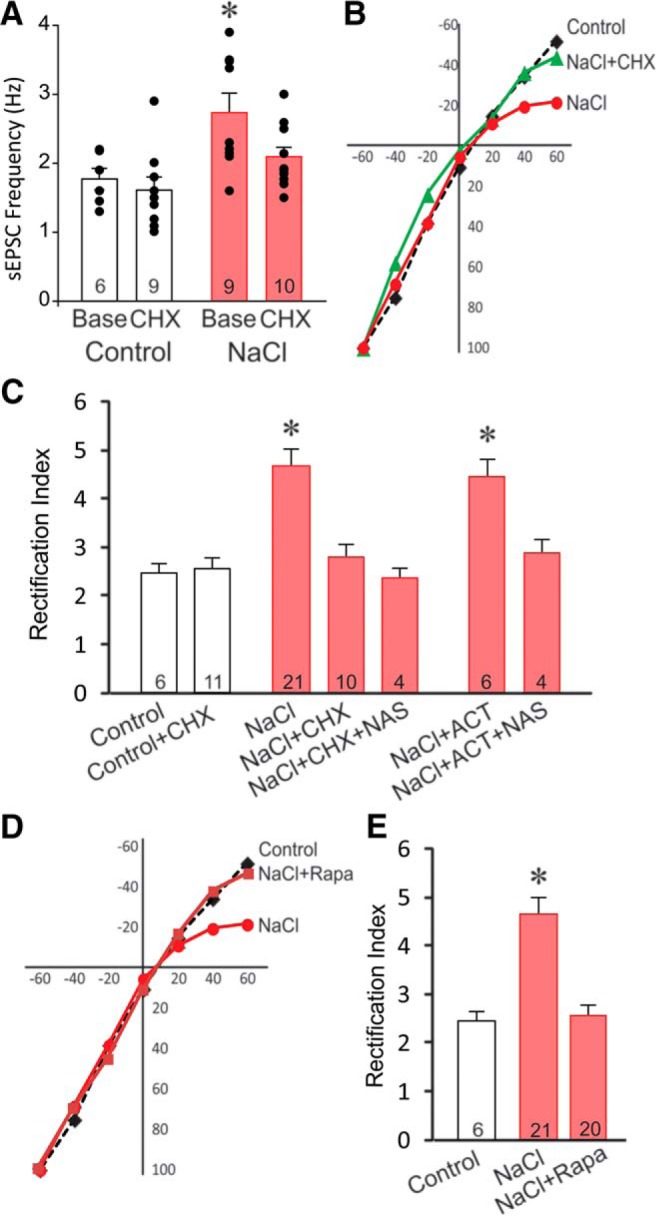
The maintenance of new inwardly rectifying AMPA currents is dependent on protein synthesis. ***A***, Blocking protein synthesis with CHX (25 μM) had no effect on sEPSC frequency in SON neurons from control rats, but reversed the increase in sEPSC frequency induced in SON neurons by salt loading to the control baseline level (one-way ANOVA followed by Dunnett’s test). ***B***, Blocking protein synthesis with CHX (NaCl+ CHX) eliminated the increase in eEPSC inward rectification seen in SON neurons from salt-loaded rats (NaCl) compared to SON neurons from control rats. ***C***, CHX had no effect on the rectification index in eEPSCs in SON neurons from control rats (control+CHX), but reversed the increase in the rectification index (NaCl+CHX) and occluded the effect of NAS (NaCl+CHX+NAS) in SON neurons from salt-loaded rats. Inhibition of gene transcription with ACT (25 μM) had no effect on the eEPSC rectification index (NaCl+ACT) and did not prevent the NAS-induced reduction in the rectification index (NaCl+ACT+NAS) in neurons from salt-loaded rats. ***D***, ***E***, Blocking mTOR activity with rapamycin (NaCl+Rapa) blocked the eEPSC inward rectification induced by salt loading and returned the eEPSC rectification index to the control level (one-way ANOVA followed by Dunnett’s test); *p < 0.05.

Dendritic protein synthesis can play a critical role in synaptic plasticity (Zukin, 2009; Cajigas et al., 2010; Swanger and Bassell, 2013; Leal et al., 2014). Increased translation of GluA1 has been linked to the increased insertion of homomeric GluA1 calcium-permeable AMPA receptors (Sutton et al., 2006; Chen et al., 2014; Stefanik et al., 2018). Interestingly, a recent study found that protein synthesis is also required for the maintenance of elevated calcium-permeable AMPA receptor levels in nucleus accumbens synapses (Scheyer et al., 2014). Therefore, we next investigated the role of rapid protein translation in the AMPA receptor plasticity seen in the chronic salt-loading rat model. In these experiments, brain slices from salt-loaded and control rats were preincubated for 1–2 h in the protein synthesis inhibitor CHX (25 μM) before performing whole-cell recordings. There was a significant difference in the effect of CHX pretreatment on sEPSCs in SON neurons (one-way ANOVA, *F*_(3,30)_ = 6.5, *p* < 0.05). *Post hoc* Dunnett’s tests revealed a significant increase in the baseline sEPSC frequency in SON neurons from salt-loaded rats compared to the baseline sEPSC frequency in control rats (NaCl baseline: 2.7 ± 0.3 Hz vs control baseline: 1.7 ± 0.2 Hz; *p* < 0.05, Dunnett’s test; Fig. 6*A*), as described above. While blocking protein synthesis with CHX pretreatment had no effect on the sEPSC frequency in SON neurons from the control rats (control CHX: 1.6 ± 0.2 vs control baseline: 1.7 ± 0.2 Hz; n.s., Dunnett’s test), CHX returned the sEPSC frequency in the SON neurons from the salt-loaded rats to a frequency not significantly different from the baseline sEPSC frequency in the control rats (NaCl CHX: 2.0 ±0.2 Hz vs control baseline: 1.7 ± 0.2 Hz, n.s., Dunnett’s test; Fig. 6*A*).

The CHX effect on AMPA-dependent eEPSC current−voltage relations also differed between the SON neurons from the control and salt-loaded groups. CHX pretreatment had no effect on the eEPSC current−voltage relationship in SON neurons from control rats, but significantly attenuated the voltage-dependent inward rectification in AMPA-mediated eEPSCs in SON neurons from salt-loaded rats (Fig. 6*B*,*C*). The CHX pretreatment decreased the AMPA current rectification index in SON neurons from the salt-loaded group (one-way ANOVA *F*_(7,74)_ = 9.258, *p* < 0.001) to a level comparable to that in SON neurons from the control group (NaCl+CHX: 2.8 ± 0.3 vs control: 2.9 ± 0.2, n.s., *post hoc* Dunnett’s test; Fig. 6*C*). Furthermore, the CHX pretreatment occluded the effect on the rectification index of NAS blockade of calcium-permeable AMPA receptors in neurons from salt-loaded rats (NaCl+CHX: 2.8 ± 0.3 vs NaCl+CHX+NAS: 2.4 ± 0.2; Fig. 6*C*), indicating that the observed reversal of the increase in rectification index by blocking protein synthesis was due to the loss of calcium-permeable AMPA receptor signaling.

We also tested for a transcription dependence of the AMPA receptor plasticity induced by chronic salt loading. Blocking gene transcription with the transcription inhibitor ACT (25 μΜ) had no effect on the inward rectification seen in SON neurons from salt-loaded rats (NaCl+ACT 4.5 ± 0.3 vs control, *p <* 0.05, *post hoc* Dunnett’s test), nor did it affect the NAS sensitivity of the AMPA receptor-mediated synaptic currents in these neurons, as NAS reduced the rectification index to a level not significantly different from the rectification index of SON neurons from control rats (NaCl+ACT+NAS 2.9 ± 0.3 vs control, n.s., *post hoc* Dunnett’s test; Fig. 6*C*). Together, these findings indicated that the increase in calcium-permeable AMPA synaptic currents in slices from salt-loaded rats is dependent on protein translation but not on gene transcription.

mTOR is a protein kinase in the PI3 kinase family that has been implicated in BDNF-dependent dendritic protein synthesis (Tang and Schuman, 2002; Takei et al., 2004). The rapid silencing of calcium-permeable AMPA receptor currents by inhibiting protein synthesis suggests a local dendritic translation mechanism. Therefore, we tested for the mTOR dependence of the maintenance of AMPA receptor plasticity following chronic salt loading by inhibiting mTOR activity with Rapa. Pre-incubation of slices for 1–2 h in Rapa (500 nM) significantly attenuated the voltage-dependent inward rectification of AMPA eEPSCs in SON neurons from salt-loaded rats (Fig. 6*D*,*E*). Rapa pretreatment decreased the AMPA current rectification index in neurons from salt-loaded rats to a value that did not differ from the rectification index of AMPA currents recorded in neurons from control rats (NaCl+Rap: 2.6 ± 0.1 vs control, n.s., *post hoc* Dunnett’s test). These results suggest that dendritic mTOR-dependent protein translation plays a critical role in the maintenance of the homomeric GluA1 AMPA receptor-containing glutamate synapses induced by chronic salt loading.

## Discussion

Sustained physiologic activation of the hypothalamic-neurohypophysial system leads to pronounced neuronal-glial remodeling and synaptic plasticity in SON and PVN magnocellular neurons (Bonfanti and Theodosis, 2009; Tasker et al., 2017). Among the plastic changes, glutamatergic synapses on magnocellular neuronal somata/proximal dendrites increase in number in response to chronic salt loading (Miyata et al., 1994b; Hatton, 1997), lactation (El Majdoubi et al., 1996), and repeated restraint stress (Miyata et al., 1994a), and glutamatergic synaptic inputs are enhanced during chronic salt loading (Di and Tasker, 2004) and lactation (Stern et al., 2000; Oliet et al., 2001). We hypothesized that this increase in excitatory synaptic innervation during salt loading would be accompanied by altered AMPA receptor expression and signaling.

The lack of significant regulation of any of the AMPA receptor subunit mRNAs detected with qPCR was unexpected and suggested that any change in glutamate receptor expression that accompanies the increased glutamate synapse numbers during salt loading must be mediated by post-transcriptional regulation of protein expression. Our Western blot analysis supported this by revealing an increase in GluA1 surface and intracellular protein, with no change in the expression of the other three AMPA receptor subunits. This indicated, therefore, that chronic salt loading regulates AMPA receptor expression at the level of protein translation, not transcription, and that it induces an increase in GluA1 subunits, but not in GluA2, GluA3, or GluA4 subunits. This in turn suggested the possibility of an increase in the relative contribution of AMPA receptors that contain GluA1 but not GluA2 subunits after salt loading (i.e., calcium-permeable AMPA receptors). Our whole-cell recordings of EPSCs confirmed an increase in the proportion of GluA1-containing, GluA2-lacking AMPA receptors at glutamate synapses induced by chronic salt loading. Salt loading resulted in an increase in the sEPSC frequency and amplitude, but did not cause a change in the glutamate release probability measured by paired-pulse analysis, consistent with our previous findings (Di and Tasker, 2004) and suggesting a change in presynaptic glutamate release sites (i.e., synapse number; Hatton, 1997; Theodosis et al., 1998). Salt loading also resulted in a robust increase in the sensitivity of pharmacologically isolated synaptic AMPA currents to NAS, a selective antagonist of GluA2-lacking, calcium-permeable receptors, and an increase in the AMPA current inward rectification at membrane potentials >0 mV, which was reversed by NAS. NAS caused a reduction in the amplitude of eEPSCs and a reduction in both the amplitude and frequency of sEPSCs in SON neurons from salt-loaded rats, while it had no effect on eEPSCs or sEPSCs in SON neurons from control rats. Since the increase in the proportion of NAS-sensitive AMPA currents in slices from salt-loaded rats may reflect the increase in glutamate synapses induced by salt loading, this suggests that the new synapses formed during chronic dehydration express predominantly calcium-permeable AMPA receptors. The strong correlation between the proportion of AMPA current blockade by NAS and the membrane capacitance of SON neurons suggests that the increase in membrane surface area is correlated with the increase in synaptic calcium-permeable AMPA receptors, again supporting the notion that the new glutamate synapses covering the added membrane surface are predominantly composed of GluA1 receptors. The most compelling evidence for the new synapses being composed of GluA1 homomeric receptors was found by blocking calcium-permeable AMPA receptors and inhibiting protein synthesis, which reversed the salt loading-induced increase in sEPSC frequency and AMPA current rectification. These observations together strongly suggest that the synapses newly formed during salt-loading are composed of GluA1 homomeric AMPA receptors, while the old glutamate synapses are composed of GluA2-containing AMPA receptors. Evidence for GluA2-containing synapses and GluA2-lacking glutamate synapses co-innervating the same neurons has been shown previously in hippocampal neurons and retinal ganglion cells (Lerma et al., 1994; Zhang et al., 1995).

Previous studies have reported that OT neurons display AMPA receptor-mediated mEPSCs with larger amplitude, faster decay kinetics and stronger inward rectification than VP neurons, which suggests that OT and VP neurons may have a different GluA2 composition in synaptic AMPA receptors (Stern et al., 1999). The increase in glutamatergic synaptic inputs to magnocellular neurons during lactation was reported to be specific to OT neurons (El Majdoubi et al., 1996; Stern et al., 2000). We were not able to distinguish between OT and VP neurons in our molecular analyses; however, we identified OT and VP neurons in our electrophysiological experiments and found no obvious difference in the effect of salt loading on AMPA currents in the two cell types. The source of this discrepancy may stem from differences in the plastic changes in glutamatergic synapses and receptors induced by different physiologic challenges (i.e., lactation vs salt loading). Indeed, we have found that lactation induces changes in AMPA receptor subunit protein expression (S. Wang and J.G. Tasker unpublished observations) which are different from those reported here that are induced by salt loading.

Studies have shown that the transient expression of calcium-permeable, GluA2-lacking receptors might provide an important route for calcium entry during synapse maturation and a possible mechanism for short- and long-term plasticity in developing networks (Rozov and Burnashev, 1999; Liu and Cull-Candy, 2000). While magnocellular neurons do not exhibit prominent dendritic spines, they show significant changes in excitatory synapse number with salt loading, and the increase in calcium-permeable AMPA receptors and resulting changes in calcium permeability of glutamate synapses during salt loading may play an important functional role in the synaptic plasticity of glutamate synapses in these cells. AMPA receptor-mediated, calcium-dependent synaptic plasticity may take the place of NMDA receptor-dependent plasticity under conditions of astrocyte retraction (e.g., during salt loading and lactation), when NMDA receptor mechanisms are diminished due to the loss of the astrocytic source of the NMDA receptor co-agonist d-serine (Panatier et al., 2006).

The change in AMPA receptor-mediated synaptic currents with salt loading was reversed by blocking protein synthesis, but was insensitive to inhibition of transcription, suggesting that continuous protein translation is required to maintain synaptic function at the new synapses formed during salt loading. This is similar to the elevation in calcium-permeable AMPA receptor-mediated currents observed in the nucleus accumbens after cocaine withdrawal, which is also reversed by inhibiting protein synthesis prior to recordings (Scheyer et al., 2014). However, while the increase in calcium-permeable AMPA receptor levels in the nucleus accumbens persists for months during cocaine withdrawal (Wolf, 2016), the new glutamate synapses in magnocellular neurons induced by salt-loading, which we show here to comprise calcium-permeable AMPA receptors, are lost within days of reversal of hyperosmotic conditions (Miyata et al., 1994b). Blocking protein synthesis silenced the calcium-permeable AMPA receptor synapses in the SON neurons from salt-loaded rats, which suggests that the AMPA receptors formed at new excitatory synapses during salt loading are highly labile.

The production of new calcium-permeable AMPA receptors during salt loading is likely to be due to local dendritic protein synthesis, since it was blocked by inhibiting mTOR activity. The silencing of the salt loading-induced excitatory synapses could be due to structural reorganization of the afferent axons and loss of synaptic contacts (i.e., presynaptic), or to the trafficking of the calcium-permeable AMPA receptors out of the synapses (i.e., postsynaptic). It is unlikely that the synaptic silencing is due to physical pruning of afferent axon terminals because the structural changes induced by salt loading take several days, not hours, to return to control levels following termination of the hyperosmotic stimulation (Miyata et al., 1994b). The silencing of the excitatory synapses with protein synthesis blockade, therefore, is likely to be the result of AMPA receptor trafficking out of the synapse. The newly formed GluA1 homomeric glutamatergic synapses, therefore, are highly labile, subject to silencing with modulation of protein synthesis and trafficking, while the older, GluA*-GluA2 heteromeric synapses are more stable.

The calcium permeability and the lability of the newly formed glutamate synapses may contribute to the state-dependent plasticity of the magnocellular system that is highly responsive to changing osmotic conditions. Glutamate receptor activation is necessary for the neuronal-glial structural plasticity induced by osmotic stimulation (Langle et al., 2003). The increase in calcium influx through the new calcium-permeable AMPA receptors, therefore, may contribute to the inductive and/or maintenance mechanism(s) responsible for the neuronal-glial structural reorganization and the synaptic facilitation seen in magnocellular neurons during chronic hyperosmotic challenge.

## Synthesis

Reviewing Editor: Quentin Pittman, University of Calgary Decisions are customarily a result of the Reviewing Editor and the peer reviewers coming together and discussing their recommendations until a consensus is reached. When revisions are invited, a fact-based synthesis statement explaining their decision and outlining what is needed to prepare a revision will be listed below.

The reviewers and I agree that your data are interesting but find a number of inconsistencies, especially in the result reporting that should be addressed. A synthesis of the reviews is provided below:

This paper reports AMPA receptor synaptic plasticity in the supraoptic nucleus (SON) after 5-7 days of salt loading. Previous studies have established that SON neurons and their synaptic inputs undergo robust plasticity following physiological challenges including salt loading. This paper characterized mRNA and protein expression profiles of GluA1-4 subunits after salt loading in rats, and reports a selective upregulation of GluA1 protein with no correlating change in mRNA. Patch clamp electrophysiology in slice preparation found that salt loading increased an inward rectification of eEPSCs at Vm &gt; 0 mV, indicating an emergence of GluA2 lacking AMPA receptors. The bath application of NAS, a pharmacological inhibitor for GluA2 lacking AMPA receptors, decreased sEPSC frequency and amplitude and reversed the rectification of eEPSCs only in slices from salt loaded rats. Similarly, inhibitors for protein synthesis and mTOR signaling reversed the effects of salt loading on sEPSCs and the rectification of eEPSCs, indicating that the increase of GluA2 lacking AMPA receptors require a continuous protein synthesis. The finding that salt loading may cause a selective increase in GluA2-lacking AMPA receptor is potentially interesting. However, there are numerous inconsistencies in data reporting (see details below), compromising the credibility of the data.

Specific comments:

1) This reader is confused by the discrepancy between df and N reported. For example, Fig. 3A reports N=12 and N=22 for control and NaCl, respectively. On the other hand, in the text, (p15) the total sample number estimated from df of two t-test (separately run for VP and OT neurons) are 33 (VP) +11 (OT) =44. This is a major difference in sample number. Another example is Fig. 4B where N=13 and N=19 for control and NaCl, respectively. However, in the text, df are 8 and 36 for control and NaCl, respectively.

2) Fig. 4C. In experiments that examine drug-induced changes in eEPSC, it is expected that both Amplitude and PPR are measured from the same set of neurons. Why N for Amp and PPR are different?

3) Fig. 3 C and E. The reader assumes that E (rectification index) was calculated from the data shown in C. Why the sample numbers are different? In C, N= 4 and N=6 for control and NaCl, respectively. In E, N=10 for control and N=9 for NaCl.

4) Fig.5 The reader assumes that this correlation was made from the data shown in Fig. 4D examining the reversal of rectification index by NAS. The reported sample numbers are N=8 and N=8 for control and NaCl, respectively. So total N=16. However, in the text (p20), N=18 is reported for testing the correlation. Even more concerning is that in Fig. 5, there are 19 data points (not N=18).

5) The correlation between synaptic plasticity and Cm is potentially interesting. The same correlation should be tested for NAS-induced sEPSC change and Cm, if these data are available

6) CHX and/or rapamycin may inhibit presynaptic glutamate release (at new synapses the authors propose), resulting in changes in EPSCs. Comment?

7) qPCR methods. The authors state “The geometric means of cytochrome C-1 andcyclophilin or of cytochrome C-1, cyclophilin and GAPDH were used to normalize gene expression”. Please specify which one was used.

8) Fig. 2C, Surface protein cross linking using BS3. M&M should provide more details about how this procedure works.

9) Fig. 6A. Two way ANOVA (NaCl x CHX) but not one way ANOVA should be used.

10) P22, line 1. Rectification index are reported in Hz.

11). The authors should consider indicating which data came from the VP-eGFP and OT-RFP transgenic rats a which data can from the Sprague-Dawley rats.

12) Fig 2B2 there is a discrepancy between the single asterisk in the figure and description in the figure legend and text.

13) in Fig 2 legend you claim: “There was no

difference between the AMPA receptor subunit S/I ratios between SON tissues from control and saltloaded

rats, suggesting no effect of salt loading on the relative membrane content of the AMPA receptor

subunits.” It would seem that the electrophysiology clearly indicates the membrane subunit content to be different. Please clarify.

## Author Response

We thank the editor and reviewer for their constructive comments and suggestions for improving our manuscript. We apologize for some of the careless mistakes we made, which we have corrected, and we have addressed all the comments from the first review in bulleted form below.

Reviewer 1:

1) This reader is confused by the discrepancy between df and N reported. For example, Fig. 3A reports N=12 and N=22 for control and NaCl, respectively. On the other hand, in the text, (p15) the total sample number estimated from df of two t-test (separately run for VP and OT neurons) are 33 (VP) +11 (OT) =44. This is a major difference in sample number. Another example is Fig. 4B where N=13 and N=19 for control and NaCl, respectively. However, in the text, df are 8 and 36 for control and NaCl, respectively.

We apologize for the confusion. Figure 3A shows data from the VP neurons only, which allowed us to clearly show the individual point distributions. We have corrected the figure legend to state that only VP neurons are represented and have moved the figure reference in the text to make this clearer. For the unpaired t test, the df = (12 control VP neurons + 22 NaCl VP neurons) - 2 = 32, hence the t32 reported in the text. The same situation applies to the OT neuron analysis, (5 control OT neurons + 7 NaCl OT neurons) -2 = 10, hence t10.

In Fig. 4B, the sample size in the control group should be 5 and in the salt-loaded group it should be 19. In the text, the df = 4 and 18, respectively, using a paired t test. We mistakenly reported performing an unpaired t test in the previous version.

2) Fig. 4C. In experiments that examine drug-induced changes in eEPSC, it is expected that both Amplitude and PPR are measured from the same set of neurons. Why N for Amp and PPR are different?

The PPR analysis was performed on a subset of the neurons used for analysis of eEPSCs (i.e., we did not perform paired stimulation on all the neurons).

3) Fig. 3 C and E. The reader assumes that E (rectification index) was calculated from the data shown in C. Why the sample numbers are different? In C, N= 4 and N=6 for control and NaCl, respectively. In E, N=10 for control and N=9 for NaCl.

The rectification index in Fig. 3E was calculated from current responses to stimulation only at Vm's of -60 mV and +40 mV, whereas the AMPA I-V curve in Fig. 3C was generated from cells that were stepped to potentials between -60 and +40 mV by 20-mV steps. The cells used for the current-voltage plot were included in the data set used to calculate the rectification index. This was not clear in the original version and has been revised for clarity.

4) Fig.5 The reader assumes that this correlation was made from the data shown in Fig. 4D examining the reversal of rectification index by NAS. The reported sample numbers are N=8 and N=8 for control and NaCl, respectively. So total N=16. However, in the text (p20), N=18 is reported for testing the correlation. Even more concerning is that in Fig. 5, there are 19 data points (not N=18).

The correlation was actually between the membrane capacitance (Cm) and the NAS-induced change in eEPSC amplitude, not the rectification index as reported in Fig 4D. The sample size was indeed 19, and not 18. We thank the reviewer for catching this mistake, which has been corrected in the revision.

5) The correlation between synaptic plasticity and Cm is potentially interesting. The same correlation should be tested for NAS-induced sEPSC change and Cm, if these data are available

We thank the reviewer for this suggestion. We conducted this analysis and found a similar correlation between the sEPSC sensitivity to NAS and the membrane capacitance. This has been added to the revised text.

6) CHX and/or rapamycin may inhibit presynaptic glutamate release (at new synapses the authors propose), resulting in changes in EPSCs. Comment?

There was no change in the release probability, which suggests a postsynaptic effect. Also, the shift in the current-voltage curve to a linear relationship indicates that this a postsynaptic effect, since it represents a change in the evoked postsynaptic response.

7) qPCR methods. The authors state “The geometric means of cytochrome C-1 and cyclophilin or of cytochrome C-1, cyclophilin and GAPDH were used to normalize gene expression”. Please specify which one was used.

This was supposed to state “the geometric mean” (i.e., singular, not plural), since the three standards were averaged and used as the reference. We apologize for the misstatement, which has been corrected.

8) Fig. 2C, Surface protein cross linking using BS3. M&M should provide more details about how this procedure works.

We have provided more details on the technique and have referenced the original paper that provides more protocol detail. We have also added some of the surface and intracellular measures that went into calculating the surface-to-intracellular ratios to Figure 2.

9) Fig. 6A. Two way ANOVA (NaCl x CHX) but not one way ANOVA should be used.

We performed the two-way ANOVA and obtained the same results. Normally, we would agree that the 2-way ANOVA is more appropriate, but we had already established the change in EPSC frequency with salt loading, and we wanted to focus here on the return of the EPSC frequency in NaCl animals to control levels by blocking protein synthesis with CHX. The comparison of each of the groups (Control-CHX, NaCl-baseline, NaCl-CHX) to the control group (Control-baseline) using the 1-way ANOVA followed by the Dunnett's test makes this very clear and easy for the reader to understand. The 2-way ANOVA compares each of the groups to each other, making the figure less focused on the reversal of the increase in sEPSC frequency in the NaCl group to the Control-Base level and more confusing (i.e., to make the point, one would have to notice in the 2-way ANOVA figure that the NaCl-CHX group is not different from the Control-Base group).

10) P22, line 1. Rectification index are reported in Hz.

This has been corrected.

11). The authors should consider indicating which data came from the VP-eGFP and OT-RFP transgenic rats a which data can from the Sprague-Dawley rats.

The molecular studies were carried out in Sprague-Dawley rats and the electrophysiological studies were performed using the VP-eGFP and OT-RFP transgenic rats, which is now indicated in the respective sections of the Materials and Methods and throughout the Results.

12) Fig 2B2 there is a discrepancy between the single asterisk in the figure and description in the figure legend and text.

This has been corrected.

13) in Fig 2 legend you claim: “There was no difference between the AMPA receptor subunit S/I ratios between SON tissues from control and salt-loaded rats, suggesting no effect of salt loading on the relative membrane content of the AMPA receptor subunits.” It would seem that the electrophysiology clearly indicates the membrane subunit content to be different. Please clarify.

The lack of change in the S/I ratio with the BS3 crosslinking approach indicates that the membrane content relative to the intracellular content did not change. The total protein content of GluA1 increased, but there was an increase in both the surface and the intracellular GluA1 protein levels in the NaCl group, such that the ratio did not change. We have added the individual measurements (surface and intracellular protein) to Figure 2 and have further clarified this in the text and figure legend.
